# Antitumoral Potential of Tunisian Snake Venoms Secreted Phospholipases A_2_

**DOI:** 10.1155/2013/391389

**Published:** 2013-01-31

**Authors:** Raoudha Zouari-Kessentini, Najet Srairi-Abid, Amine Bazaa, Mohamed El Ayeb, Jose Luis, Naziha Marrakchi

**Affiliations:** ^1^Laboratoire des Venins et Biomolecules Therapeutiques, Institut Pasteur de Tunis, 13, Place Pasteur, 1002 Tunis, Tunisia; ^2^Université de Tunis el Manar, 1068 Tunis, Tunisia; ^3^Centre de Recherche en Oncologie Biologique et Oncopharmacologie (CRO2), INSERM UMR 911, Marseille, France; ^4^Université d'Aix-Marseille, Marseille, France; ^5^Faculté de Médecine de Tunis, 1007 Tunis, Tunisia

## Abstract

Phospholipases type A_2_ (PLA_2_s) are the most abundant proteins found in Viperidae snake venom. They are quite fascinating from both a biological and structural point of view. Despite similarity in their structures and common catalytic properties, they exhibit a wide spectrum of pharmacological activities. Besides being hydrolases, secreted phospholipases A_2_ (sPLA_2_) are an important group of toxins, whose action at the molecular level is still a matter of debate. These proteins can display toxic effects by different mechanisms. In addition to neurotoxicity, myotoxicity, hemolytic activity, antibacterial, anticoagulant, and antiplatelet effects, some venom PLA_2_s show antitumor and antiangiogenic activities by mechanisms independent of their enzymatic activity. This paper aims to discuss original finding against anti-tumor and anti-angiogenic activities of sPLA_2_ isolated from Tunisian vipers: *Cerastes cerastes* and *Macrovipera lebetina*, representing new tools to target specific integrins, mainly, *α*5*β*1 and *α*v integrins.

## 1. Introduction 

Snake venom is a natural biological resource, containing several neurotoxic, cardiotoxic, cytotoxic, and many other different active compounds [1, 2]. Due to this broad range of biological functions, these biomolecules have been the subject of hundreds of scientific articles in different research fields, including biochemistry, biophysics, pharmacology, toxicology, and medicine [2–5]. Viperidae snake venoms contain class II PLA_2_s, which share structural features with secreted PLA_2_ (sPLA_2_) of the class II-A present in inflammatory exudates in mammals. A number of venom PLA_2_s have been shown to induce a variety of pharmacological effects although comprehensive studies of the actions of venom PLA_2_s in the various events of toxicity are scarce [6, 7].

## 2. Viperidae Snake Venom Phospholipase A_2_ Enzymes: Secreted Phospholipases A_2_

Secreted PLA_2_ constitute a large superfamily of enzymes that are widely distributed in living organisms. The sPLA_2_ from Viperidae snake venoms fall under group II. They are generally Ca^2+^-dependant enzymes that catalyze the hydrolysis of the sn-2 fatty acid bond of phospholipids to release free fatty acids and lysophospholipids [7]. These enzymes are small proteins (~13-14 kDa), containing 120–125 aminoacid residues, 7 disulfide bridges, and have a partially conserved structure that define the PLA_2_ fold [8]. Group II snake venom PLA_2_ enzymes can also be divided into different subgroups on the basis of the aminoacid residue in the forty-ninth position. Asp49 plays an important role in catalysis and it is conserved in most snake venom PLA_2_ enzymes, and hence these are identified as D49 enzymes [9]. However, in some of the group IIA PLA_2_ enzymes this aminoacid residue is replaced by lysine, serine, asparagine, or arginine and they are identified as K49 [10], S49 [11], N49 [12], or R49 [13] enzymes, respectively. Substitution of Asp in the forty-ninth position interrupts the binding of cofactor Ca^2+^ to the Ca^2+^-binding loop, and hence “mutants” show low or no hydrolytic activity [10, 14, 15]. In addition, there are several substitutions in the Ca^2+^-binding loops of these mutant enzymes.

Secreted phospholipases A_2_ constitute major components of snake venoms and have been extensively investigated not only because they are very abundant in these venoms but mainly because they display a variety of relevant toxic actions such as neurotoxicity, myotoxicity, cytotoxicity, cardiotoxicity, edema-inducing, artificial membrane disrupting convulsant, hypotensive and proinflammatory effects [7, 16–19]. Besides, they exert a wide range of biological effects, including anticoagulant, platelet aggregation inhibiting [7, 20, 21], bactericidal [22], anti-HIV [23], antimalarial and anti-parasitic [24], antitumor [21, 25, 26], and recently anti-angiogenic effect [27–29]. Due to this functional diversity, these structurally similar proteins aroused the interest of many researchers as molecular models for study of structure-function relationships. One of the main experimental strategies used for the study of myotoxic PLA_2_s is the traditional chemical modification of specific aminoacid residues and examination of the consequent effects upon the enzymatic, toxic, and pharmacological activities. Furthermore, some venom sPLA_2_ have no catalytic activity while they exert various toxic and pharmacological effects [17, 21, 26]. The absence of direct correlation between catalytic activity and pharmacological effects has led to the hypothesis that specific actions of sPLA_2_ are due to the presence of pharmacological sites on the sPLA_2_ surface overlapping or distinct from the catalytic site. These pharmacological sites would allow the sPLA_2_ to bind specifically to soluble or membrane-bound proteins that participate to the sPLA_2_ mechanism of action [30].

Since this hypothesis was proposed, a collection of binding proteins have been identified using several toxic snake venom sPLA_2_ [31]. Besides *β*-bungarotoxin [32, 33], early studies with the neurotoxic sPLA_2_ OS2 from Australian Taipan snake *Oxyuranus scutellatus scutellatus* have led to the identification of two families of binding proteins called N- and M-type receptors [31, 34, 35]. The N-type receptors are present in mammalian brain and other tissues. Neurotoxic sPLA_2_, such as OS2, bind with N-type receptors with high affinity, while nontoxic sPLA_2_ including OS1 bind with much lower affinity, suggesting that these receptors are involved in neurotoxicity.

Conversely, the M-type receptors bind with high affinity both toxic and nontoxic sPLA_2_ including OS1 and OS2 [31]. Importantly, the M-type receptors also bind with several mammalian sPLA_2_ [31, 36], suggesting that these proteins are the endogenous ligands for these receptors, and possibly for the collection of binding proteins initially identified with venom sPLA_2_.

## 3. Tunisian Viperidae Snake Venom Proteins

Snake venom is a natural source for molecules known as modulators of integrin-mediated functions [37]. Pharmacological study of snake venoms reveals structural and functional polymorphisms of proteins they contain. In our laboratory in Pasteur Institute of Tunis, we are interested in studying different pharmacological effects of Tunisian Viperidae venoms, mainly, the horned viper, *Cerastes cerastes*, *Macrovipera lebetina transmediterranea,* and *Cerastes vipera* [38]. Bazaa et al. showed that these venoms contain proteins belonging to a few protein families. However, each venom showed distinct degree of protein composition complexity. The three venoms shared a number of protein classes though the relative occurrence of these toxins was different in each snake species. On the other hand, the venoms of the *Cerastes* species and *Macrovipera lebetina* each contained unique components [38]. The comparative proteomic analysis of Tunisian snake venoms provides a comprehensible catalogue of secreted proteins, which may contribute to a deeper understanding of the biological effects of the venoms and may also serve as a starting point for studying structure-function correlations of individual toxins.

Thereby, disintegrins and C-type lectins (CLPs) are among the most studied proteins proved to be components of medical and biotechnological value [39–42]. Indeed, they are potent and specific antagonists of several integrins, such as  *α*v*β*3 and  *α*5*β*1 [43, 44] and can thus act in many biological processes including platelet aggregation, angiogenesis, tumor invasion, and bone destruction [39, 45–47]. On the other hand, CLPs were first described as modulators of platelet before their antiadhesive activity was highlighted [48–50]. CLPs are thus able to inhibit integrin-dependent proliferation, migration, invasion, and angiogenesis [26, 44, 51, 52]. Sarray and coworkers have isolated lebectin and lebecetin, two C-type lectins, from *Macrovipera lebetina* snake venom inhibiting  *α*5*β*1- and  *α*v-containing integrins [43, 44]. Since their initial characterization, snake venom disintegrins have been extensively studied [39, 46], they are potent inhibitors of integrin-ligand interactions. The integrin inhibitory profile of disintegrins primarily depends on the sequence of a tripeptide located at the apex of a mobile loop and constrained in its active conformation by the appropriate pairing of disulfide bonds. So, CC5 and CC8 have been previously characterized as *Cerastes cerastes* dimeric disintegrins targeting *α*_IIb_ *β*_3_,  *α*v*β*3, and  *α*5*β*1 integrins [53]. In addition to dimeric disintegrins, *Macrovipera lebetina* venom includes short disintegrin, namely, lebestatin which targets  *α*1*β*1 integrin [46].

**Figure 1:**
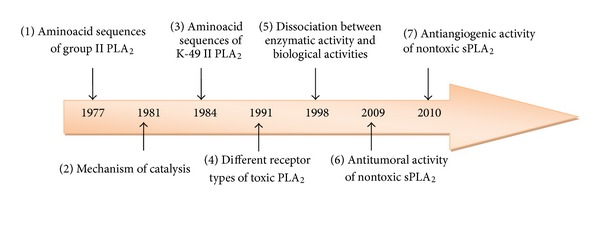
Milestones in PLA_2_ enzyme research. (1) Heinrikson et al. [81], (2) Lambeau et al. [56], (3) Maraganore et al. [10], (4) Lambeau et al. [82], (5) Landucci et al. [83], (6) Zouari-Kessenti et al. [21], Bazaa et al. [27], and (7) [27, 28].

Recently, phospholipases A_2_ (PLA_2_s, EC 3.1.1.4) have been demonstrated to modulate integrins which are essential protagonists of the complex multistep process of angiogenesis, the major target for the development of anticancer therapies [21, 27, 28] (Figure 1).

## 4. Secreted Phospholipases A_2_ from Tunisian Vipers

**Figure 2:**
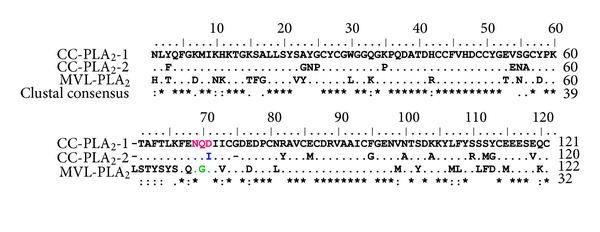
Sequence alignment of Tunisian Viperidae sPLA_2_: CC-PLA_2_-1 (ACO92622) [28], CC-PLA_2_-2 (ACO92623) [28], and MVL-PLA_2_ (CAR40186) [26]. Gaps (—) have been introduced to optimize alignment.

Three acidic, nontoxic, Asp49 phospholipases A2 have been isolated from Tunisian vipers: CC-PLA_2_-1 and CC-PLA_2_-2 from *Cerastes cerastes*, and MVL-PLA_2_ from *Macrovipera vipera*. They have a molecular weight of 13737.52, 13705.63, and 13626.64 Da, respectively. They contain, respectively, 121, 120, and 122 aminoacids, including 14 cysteines each [21, 26]. The sequences alignment shows similarity as high as 50% (Figure 2). Furthermore, none of the three PLA2s is cytotoxic up to 2 *μ*M.

CC-PLA_2_-1 and CC-PLA_2_-2 present a high enzymatic activity [21], while MVL-PLA_2_ shows a low one. Although they differ greatly in their catalytic properties, these shared many pharmacological activities proving the lack of correlation between enzymatic and pharmacological activities.

## 5. Pharmacological Activities of sPLA_2_ from Tunisian Vipers

CCPLA_2_-1, CC-PLA_2_-2, and MVL-PLA_2_ show many pharmacological effects [21, 26]. The most interesting are the antitumor and antiangiogenic activities which involve integrins [27, 28].

### 5.1. Tunisian Viperidae sPLA_2_ Effects on Haemostatic System

Snake venom toxins are now regularly used in laboratories for assaying haemostatic parameters and as coagulation reagents [54, 55]. PLA_2_ enzymes are known to inhibit blood coagulation. Depending on the dose required to inhibit coagulation, they are classified into strong, weak, and nonanticoagulant enzymes [56, 57]. Strong anticoagulant PLA_2_ enzymes inhibit the activation of FX to FXa by both enzymatic and nonenzymatic mechanisms and inhibit the activation of prothrombin to thrombin by nonenzymatic mechanism [58, 59]. In our case, 0.14 *μ*M of both CC-PLA_2_s completely inhibited plasma coagulation. Thus, CC-PLA_2_s could be considered among the most anticoagulant yet described for PLA_2_s snake venom [21]. Lizaro and coworkers showed that myotoxin II, a basic PLA_2_ from *Bothrops nummifer*, was unable to inhibit coagulation of the platelet-poor plasma until 3.57 *μ*M [60]. Moreover, it has been shown that BaspPLA(2)-II, an acidic, Asp49 PLA_2_ from *Bothrops asper* venom lacks anticoagulant activity [61].

Platelet aggregation plays a role in clot retraction and wound healing. Any alteration in platelet aggregation could lead to debilitation or death. CC-PLA_2_-1 and CC-PLA_2_-2 showed high antiplatelet aggregation activities induced by arachidonic acid or ADP [21], contrary to b/D-PLA2 which displays high enzymatic and anticoagulant activities but has no platelet aggregation [62]. Moreover, Kashima and coworkers reported that BthA-I-PLA_2_, a nontoxic acidic PLA_2_ from *Bothrops jararacussu* snake venom, inhibited ADP-induced platelet aggregation with moderate effect [63]. While, OHVA-PLA_2_, an acidic PLA_2_ from *Ophiophagus hannah*, strongly inhibited platelet aggregation in the presence of ADP or arachidonic acid [64]. It thus appears that PLA2 platelet activity is not directly due to its acidic nature or its anticoagulation activity.

### 5.2. Tunisian Viperidae sPLA_2_ Effects on Tumor Cell Behavior

Snake venom sPLA_2_ present a wide range of pharmacological effects [7], including cytotoxicity on tumor cells [7, 63, 65]. Concerning CC-PLA_2_-1, CC-PLA_2_-2, and MVL-PLA_2_, concentrations up to 2 *μ*M during 4 days did not induce detectable cytotoxicity on human cell lines IGR39 (melanoma) and HT1080 (fibrosarcoma) [21, 26].

Adhesion and cell migration are two fundamental steps in numerous diseases, like cancer. CC-PLA_2_-1, CC-PLA_2_-2, and MVL-PLA_2_ inhibit adhesion and migration of human HT1080 and IGR39 cells to fibrinogen and fibronectin. This effect persists even after complete blockage of the catalytic activity suggesting that, contrary to Bth-A-I-PLA_2_ whose antitumoral effect appears to be linked to enzymatic site [63], the inhibitory and enzymatic activities are supported by different sites. RVV-7, a cytotoxic basic PLA_2_ from *Russsell's viper* venom, inhibits also tumor development [65]. On the contrary, b/D-PLA_2_ represents the exception of these enzymes as it stimulates tumor growth [62]. Since Tunisian phospholipases A_2_ are not cytotoxic, it seems that their antitumoral activity is exerted by a different mechanism. Using different assays, such as a solid-phase binding assay and a panel of immobilized antibodies, we have proved that CC-PLA_2_-1, CC-PLA_2_-2, and MVL-PLA_2_ inhibit cell adhesion and migration by interacting directly with  *α*v and  *α*5*β*1 integrins [26, 28].

### 5.3. Tunisian Viperidae sPLA_2_ Effects on Angiogenesis

Angiogenesis is fundamental to normal healing, reproduction, and embryonic development. However, this process is also important in the pathogenesis of a broad range of disorders such as arthritis and cancer [66]. Angiogenesis is thus required to sustain malignant cells with nutrients and oxygen for tumors to grow beyond a microscopic size. Thus, the microvascular endothelial cell recruited by a tumor is an important target in cancer therapy and has the advantage of being genetically stable. Therefore, treating both the cancer cell and the endothelial cell in a tumor may be more effective than treating the cancer cell alone.

The role of  *α*v*β*3 integrin in the angiogenic process is well documented [67]. In the last decade, several clinical trials evaluating the efficacy of  *α*v*β*3 blockers have led to encouraging results in cancer therapy and diagnosis. Similarly,  *α*5*β*1 integrin is involved in angiogenesis and more precisely in growing vessels, but its expression disappears in mature vessels [68]. Thereby, when tested *in vitro*, the two CC-PLA_2_ and MVL-PLA_2_ impaired adhesion and migration of HBMEC (human brain microvascular endothelial cells) and HMEC-1 (human microvascular endothelial cell), respectively, by interfering with integrin function. Moreover, using the CAM assay, an *ex vivo* model, these sPLA_2_ strongly reduced vasculature development. The treatment reduced the number of new capillaries and branching, without affecting the mature blood vessels, suggesting once again the implication of  *α*5*β*1 integrin. Interestingly, CC-PLA_2_-1 and CC-PLA_2_-2 inhibit spontaneous angiogenesis as well as angiogenesis induced by growth factors such as VEGF or bFGF [28]. The antiangiogenic effect of PLA_2_ can be due partly to the blockage of the  *α*v*β*3 and  *α*5*β*1 integrins functions. However, inhibition of angiogenesis can also result from blockage of VEGF or its receptor. Thus, it has been reported that inactive PLA_2_ homologues, such as KDR-bp isolated from *Eastern cottonmouth* venom, are common antagonists of KDR, a VEGF receptor [69].

Focal adhesions are specialized sites of attachment of cells where integrins receptors, such as  *α*v*β*3, link the extracellular matrix to the actin cytoskeleton, allowing migration [70]. Cell migration is a complex cellular behavior that results from the coordinated changes in the actin cytoskeleton and the controlled formation and dispersal of cell-substrate adhesion sites. While the actin cytoskeleton provides the driving force at the cell front, the microtubule network assumes a regulatory function in coordinating rear retraction. The polarity within migrating cells is further highlighted by the stationary behavior of focal adhesions in the front and their sliding in trailing ends [71].

Treatment of HMEC-1 cells with MVL-PLA_2_ induced important changes in cell morphology. Treated cells have a circular shape and actin stress fibers are thinner or absent, with the actin mainly located at the cell periphery. Moreover, MVL-PLA_2_ leads to drastic reduction in the size of focal adhesions and their distribution all over the ventral surface of cells, consistent with a decrease in  *α*v*β*3 integrin clustering and its absence from lamellipodia [27]. Therefore, it appears that the inhibition of migration is associated with important reorganization of the actin cytoskeleton and focal adhesions. Again, there is a clear dissociation between the anti-angiogenic effect and the catalytic activity.

Furthermore, MVL-PLA_2_ strongly increased MT dynamicity in HMEC-1 cells. Because the microtubule cytoskeleton is essential in the orchestration of endothelial cell motility [72, 73], microtubule-targeting agents are known to have antiangiogenic effects through the modulation of cytoskeleton dynamicity [27]. Thus, microtubule-binding drugs are widely used in cancer chemotherapy and also have clinically relevant antiangiogenic and vascular-disrupting properties [74].

## 6. Importance of the Identification of Pharmacological Sites

The pharmacological sites of PLA_2_ enzymes determine the affinity between the PLA_2_ and target proteins. The identification of pharmacological sites helps in (1) understanding the structure-function relationships of PLA_2_ enzymes, (2) developing strategies to neutralize the toxicity and pharmacological effects by targeting these sites, and (3) developing prototypes of novel research tools and pharmaceutical drugs [7, 8].

In our studies, we showed that CC-PLA_2_-1, CC-PLA_2_-2, and MVL-PLA_2_ target the  *α*5*β*1 and  *α*v integrins, particularly  *α*v*β*3. Moreover, angiogenesis involves expression of the later, which binds to RGD-containing components of the interstitial matrix [75].

To further understand the mechanism of action, we report that endothelial cells are able to adhere on immobilized MVL-PLA_2_ and that this adhesion is impaired by RGD peptides [27]. This suggests that interaction between MVL-PLA_2_, CC-PLA_2_-1, or CC-PLA_2_-2 and integrins involves RGD-like sequence which may be responsible for the inhibition of integrin function. This hypothesis is supported by Ramos and coworkers' study, showing that general folding of electrostatic potential is the main intervening of disintegrin-integrin interaction [76].

When MVL-PLA_2_ contains a NGD sequence, which could be considered as an RGD-like motif, CC-PLA_2_-1 and CC-PLA_2_-2 present NQD and NQI, respectively, that may also be responsible for the inhibition of integrin function.

Therefore, bioinformatics study and structural criteria that would allow identifying biologically active RGD-sites on the base of a protein's spatial structure may become a helpful tool for analysis of cellular function of proteins [77]. Furthermore, conformation of the integrin-binding loop in a protein is defined not only by physicochemical properties and conformation of the sequence itself, but also by its structural environment and therefore of the potential biological activity. Besides the RGD-like sequence site should be placed on a loop or a beta-turn to be well exposed. We can cite disintegrin, like applied model, in which we can note a loop accessible stabilized by disulfide bridges [78].

## 7. Molecular Modeling of CC-PLA_2_-1, CC-PLA_2_-2, and MVL-PLA_2_

In order to examine the site of the suspected RGD-like sequence, using the SWISS-MODEL Workspace (http://swissmodel.expasy.org/), we have determined the three-dimensional models of CC-PLA_2_-1, CC-PLA_2_-2, and MVL-PLA_2_.

**Figure 3:**
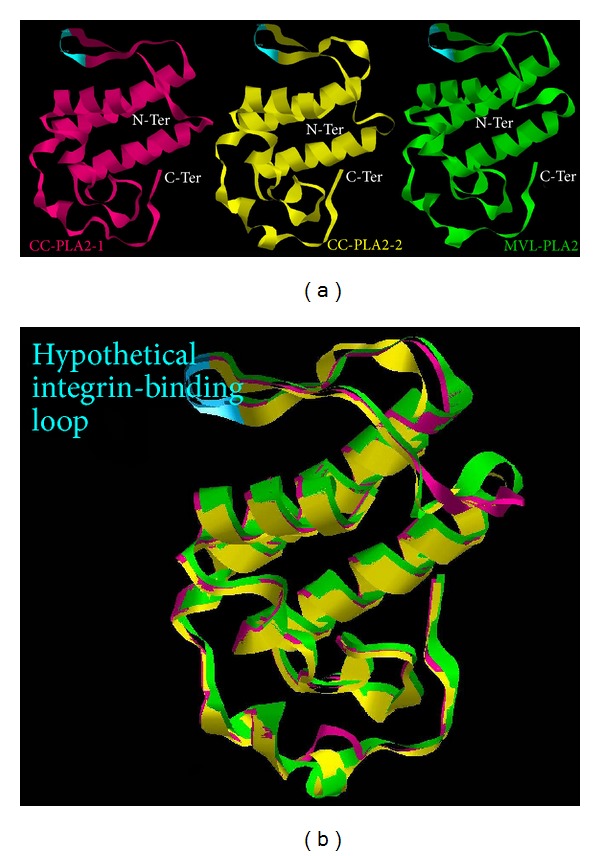
(a) Tertiary model structure of CC-PLA_2_-1 (pink), CC-PLA_2_-2 (yellow), and MVL-PLA_2_ (green). (b) Superimposition of the structural models of CC-PLA_2_-1, CC-PLA_2_-2, and MVL-PLA_2_. The hypothetical integrin-binding loop (blue) is presented in each PLA_2_.

Firstly, as shown in Figure 3(a), the three models are very similar. Interestingly, we can note the presence of very-well-exposed loop containing the suspected RGD-like motif. This loop is very similar to that of the disintegrins.

In the case of MVL-PLA_2_, we find the NGD motif, while for CC-PLA_2_-1 and CC-PLA_2_-2 there is NQD and NQI, respectively. According to our hypothesis, the residue R in RGD motif is replaced by the N which is hydrophilic and polar residue, it is even more hydrophilic than R residue, this leads to higher affinity towards the  *α*v*β*3 integrin [79]. Besides, the D residue favors recognition of  *α*v*β*3 and  *α*5*β*1 integrins [79]. In addition, in CC-PLA_2_-1 and CC-PLA_2_-2 the RGD-like motif is flanked by two E residues, highly polarized which could enhance the inhibitory effect towards integrins that bind to ligands through RGD sites, including the fibronectin receptor, mainly, the  *α*5*β*1 integrin [80].

On the other side, based on the study of disintegrins, it is known that integrin-binding ability is apparently more related to the Cys-rich domain. Similarly, CC-PLA_2_-1, CC-PLA_2_-2, and MVL-PLA_2_ present 14 Cys forming 7 disulfides bridges. We can postulate that disulfide bonds, especially Cys50–Cys86 and Cys57–Cys79, stabilized the hypothetical integrin-binding loop. The superimposition of the structural models of CC-PLA_2_-1, CC-PLA_2_-2, and MVL-PLA_2_ shows that they share similar conformational features (Figure 3(b)).

Nevertheless, further structure-function relationships study must be carried to verify this hypothesis.

## 8. Conclusion

Secreted phospholipase A_2_ enzymes, especially from *Viperidae* Snake venom, exhibit a wide variety of pharmacological effects despite their structure similarity. These enzymes provide a great challenge to protein chemists as subtle and complex puzzles in structure-function relationship. A better understanding will contribute to our knowledge of protein-protein interactions, protein targeting, and protein engineering, and to the development of better-targeted delivery systems. Further research in identifying target proteins will bring details on the mechanisms of the pharmacological effects at the cellular and molecular levels. Studies in these areas will result in new, exciting, and innovative opportunities in the future, both in finding answers to the toxicity of PLA_2_ enzymes and could bring useful tools for developing proteins with novel functions.

Interestingly, we have demonstrated that two isoforms of PLA_2_ (CC-PLA_2_-1 and -2), from horned Tunisian viper *Cerastes cerastes* and another from *Macrovipera lebetina* MVL-PLA_2_ target integrins, a large and very important family of adhesion molecules that promote stable interactions between cells and their environment [26, 28]. Indeed, these sPLA_2_ exhibit a potent antitumor and antiangiogenic activities. We showed that their effect is likely due to the inhibition of  *α*5*β*1- and  *α*v-containing integrins [26, 28].

These nontoxic secreted phospholipase A_2_ could be new tools to disrupt different steps of tumor and angiogenesis progression through integrins. It is noteworthy that this effect is independent of the enzymatic activity. This finding may serve, on the one hand, as a mean to discuss the molecular regions involved in recognition of tissue targets and, on the other hand, as starting point structure-function relationship studies leading to design a new generation of anticancer drugs.

## Abbreviations

sPLA_2_: Secreted phospholipase A_2_
CLP: C-type lectin protein
VEGF: Vascular endothelial growth factor
bFGF: Basic fibroblast growth factor
CAM: Chick chorioallantoic membrane.
